# Expanding school wellness policies to encompass the Whole School, Whole Community, Whole Child model

**DOI:** 10.3389/fpubh.2023.1143474

**Published:** 2023-03-30

**Authors:** Marlene B. Schwartz, Sandra M. Chafouleas, Jessica B. Koslouski

**Affiliations:** ^1^Rudd Center for Food Policy and Health, Department of Human Development and Family Sciences, University of Connecticut, Hartford, CT, United States; ^2^Collaboratory on School and Child Health, Neag School of Education, University of Connecticut, Storrs, CT, United States

**Keywords:** school health, wellness policies, WSCC model, nutrition, physical activity, behavioral health

## Abstract

Schools influence children’s developmental outcomes across multiple domains, including academic, social, emotional, behavioral, and physical. School district wellness policies are powerful mechanisms that set clear expectations for health-related practices in school buildings and the surrounding community. A current challenge is that many health-related school policies are narrow, siloed, and reactive instead of proactive. In this paper, we: (a) describe how written food, nutrition, and physical activity district and state policies were strengthened in the United States in response to specific concerns about childhood obesity; (b) present how schools have historically addressed policies concerning children’s social, emotional, and behavioral health; and (c) propose using the Whole School, Whole Community, Whole Child (WSCC) model to strengthen the coordination and integration of school wellness policies. We conclude by describing recently developed tools to assist school districts in implementing the WSCC model. The Wellness School Assessment Tool (WellSAT) WSCC is a quantitative measure that districts can use to code their current written policies for alignment with the WSCC model. The WSCC Policy and Practice Blueprints are action planning tools that lead school and district leaders through a series of activities to strengthen the implementation of coordinated and integrated policies and practices. By using the WSCC model and accompanying implementation tools, schools can support the development of the whole child.

## 1. Introduction

Multiple developmental pathways contribute to a child’s overall well-being, including academic, social, emotional, behavioral, and physical (1). The school setting provides a powerful opportunity to support a range of behaviors that promote student development (2). The role of local school wellness policies is to endorse or require specific practices within a school district that promote optimal health, safety, and learning (3). Because no single policy document can address the breadth of actions needed to promote all aspects of a child’s well-being, a systematic “whole child” approach to school wellness policies is needed.

In this paper, we describe the history of school-based policies designed to promote student wellness beginning with the history of local district wellness policies in the United States that emerged from a focus on childhood obesity. Next, we share parallel efforts related to social, emotional, and behavioral health, and highlight some of the challenges in creating policies to address these domains of child development. We then discuss the Whole School, Whole Community, Whole Child (WSCC) model (2). We end with the rationale for the expansion of school wellness policies to align with the WSCC model and offer resources to guide targeted efforts at strengthening the coordination and integration of school policies.

## 2. History of wellness-related school policies in the United States

### 2.1. Food, nutrition, and physical activity policies

The dramatic increase in the prevalence of childhood obesity observed between the 1970s and the 1990s prompted a cascade of activities across sectors (4). An early target was the school nutrition and physical activity environment, which was determined to be a critical setting that can support—or undermine—children’s diet quality and physical health (5). A range of efforts among public health advocates, government agencies, and policymakers aimed to improve the school environment through policies such as those that promoted healthier school meals, limited the sale of unhealthy competitive beverages and snack foods (i.e., those sold outside of the meal program), increased walking and biking to school, and strengthened physical education programs (6).

The concept of district-level school wellness policies was introduced in the United States in the 2004 WIC Reauthorization Act (7). This federal regulation stated that by 2006, all Local Education Agencies (LEAs; typically school districts) participating in any of the USDA’s federal food programs were required to convene a committee representing key groups (e.g., staff, families, and community partners) to create a school board-approved policy that included: goals for nutrition education; assurance that all federal nutrition standards were implemented; nutrition standards for competitive foods; goals for physical activity; and an evaluation plan. This strategy was notable because it did not mandate any specific local policy language; instead, it required schools to focus on the issue and articulate their own policies to improve their nutrition and physical activity environment. In 2010, the Healthy Hunger-Free Kids Act significantly strengthened the federal nutrition requirements for school meals and competitive foods and added new requirements for wellness policies (8). Among the new components was a prohibition of in-school food marketing of foods that do not meet nutrition regulations in schools and a required triennial district self-assessment of the strength and implementation of the wellness policy with a public-facing report (3).

Researchers have found that almost all US school districts have complied with this legislation and have created written wellness policies (9). Evaluations of strength and comprehensiveness indicate that policies have improved over time; however, there is still considerable variability across districts (10). Some policies merely meet the federal requirements, while others are exceptionally strong and reflect a cohesive effort to transform the school food and activity environment to maximize students’ diet quality and physical fitness. Local wellness policy strength matters: district policies that include clear, strong language about how they plan to report, monitor, and evaluate their wellness activities are significantly more likely to engage in these practices (11), and better implementation of nutrition policies has been predictive of healthier BMI trajectories (12). Research on state-level school nutrition policies suggests that strong state laws are also important, and have a positive impact on school nutrition environments, student diet quality, and student BMI (13).

As a likely consequence of the combination of federal, state, and local policy changes, the nutritional quality of school meals has improved significantly over the past decade (14) and the dietary quality among students who eat school lunch has also improved (15). In fact, school meals now provide the healthiest food most American children consume over the day (16), and the improvements due to the Healthy Hunger-Free Kids Act are associated with a significant decrease in the risk of obesity among low-income children (17). Although concerns about childhood obesity continue, progress has been made in improving the school environment to promote student wellness in the domains of nutrition, and to some extent, physical activity.

### 2.2. Social, emotional, and behavioral-focused policies

The landscape of school policies in social, emotional, and behavioral domains in the United States is less clear than those targeting physical activity and nutrition. Although some parallel work has occurred, two issues pose challenges to understanding the social, emotional, and behavioral policy landscape: (a) a lack of clear definitions of the boundaries of social, emotional, and behavioral policies and (b) a pattern of reactionary policies that do not explicitly address proactive wellness efforts.

Unlike the reasonably well-defined domain of “nutrition environment and services,” the terms “social, emotional, and behavioral” include a broad and interrelated set of features representing how we connect, how we feel, and how we act. For example, education policies that encompass social, emotional, and behavioral domains cover topics including school discipline, school climate, social–emotional learning, and trauma-informed schools (18).

A model school climate policy developed by the state of Connecticut demonstrates this breadth and complexity. It requires districts to (a) develop a shared vision and plan for promoting, enhancing, and sustaining a positive school climate; (b) develop policies that promote social, emotional, ethical, civic, and intellectual learning as well as systems that address barriers to learning; (c) implement practices that promote the learning and positive social, emotional, ethical, and civic development of students and student engagement as well as addressing barriers to learning; (d) create an environment where all members are welcomed, supported, and feel safe in school: socially, emotionally, intellectually, and physically; and (e) develop meaningful and engaging practices, activities, and norms that promote social and civic responsibilities and a commitment to social justice (19). Although it is useful to have flexibility in determining setting-specific policies and practices appropriate for the unique characteristics of different school environments, this example highlights the challenge of determining precisely which practices fall within the scope of wellness-related policies in social, emotional, and behavioral domains.

The second challenge to understanding policies related to social, emotional, and behavioral domains is that they are often reactive instead of proactive in addressing positive child development. As one example, in the U.S. Department of Education provides school climate and discipline resources to enable welcoming, supportive, and safe schools and classrooms (20). Although the available webpages provide practice tools that promote proactive and positive practices, the policy guidance is focused on eliminating disproportionality in school discipline (20). This is important but reactionary.

The current landscape of state discipline laws and regulations also emphasizes reactions to risky behaviors, such as drug possession on school grounds. A recent compilation of state discipline laws and regulations includes a range of topics: in-school discipline; conditions on use of certain forms of discipline (e.g., corporal punishment); exclusionary discipline; discipline addressing specific code of conduct violations (e.g., chronic absenteeism, dating and relationship violence); prevention, behavioral intervention, and supports; monitoring and accountability; and partnerships between schools and law enforcement (21). Again, the policy language is reactive instead of focused on positive actions to support wellness in the social, emotional, and behavioral domains.

Recently, the COVID-19 pandemic has garnered attention for policies focused on wellness promotion in social, emotional, and behavioral domains. The American Rescue Plan Act of 2021 dedicated $123 billion to promote evidence-based and culturally affirming social and emotional learning, educator well-being, and coordination across families, schools, and communities (22). It remains to be seen if positive and proactive approaches will be codified into policies that replace the current reactive ones. This shift, along with clear definitions of preventative approaches, may facilitate greater coherence within these domains of the school wellness policy landscape.

## 3. A call for better integration of child wellness efforts

**Figure 1:**
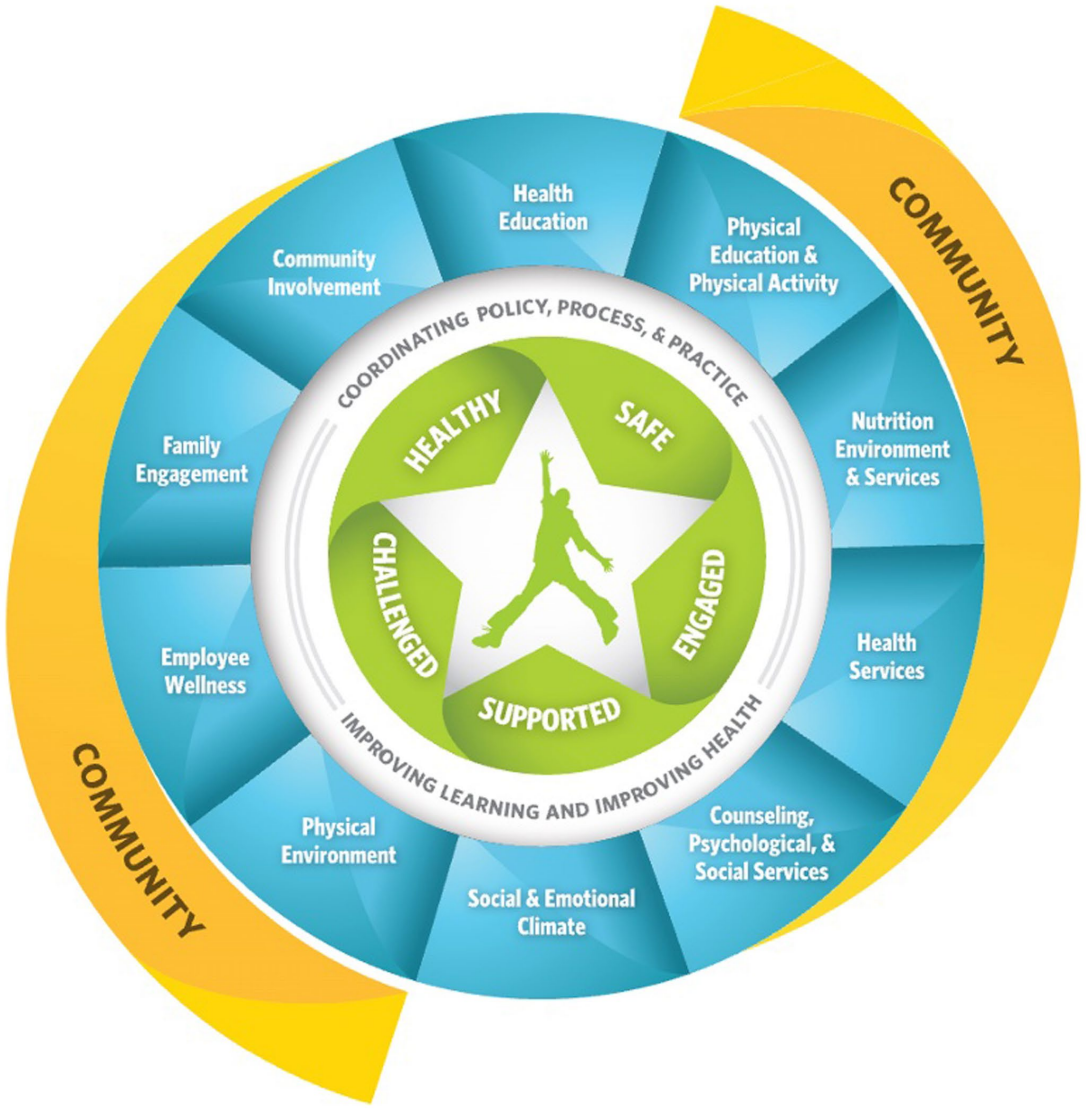
The whole school, whole community, whole child model. Available at: https://www.cdc.gov/healthyschools/wscc/index.htm.

As illustrated in Figure 1, the Whole School, Whole Community, Whole Child (WSCC) model (2), launched by the Centers for Disease Control (CDC) and ASCD in 2014, offers a framework for organizing these efforts. The WSCC model includes 10 domains: health education; physical education and physical activity; nutrition environment and services; health services; social and emotional climate; counseling, psychological, and social services; physical environment; employee wellness; family engagement; and community involvement. The WSCC model advocates for the integration and coordination of policies, processes, and practices across these domains to foster students who are healthy, safe, engaged, supported, and challenged.

## 4. Challenges and opportunities to integrate efforts

There is considerable enthusiasm for the WSCC model at the federal level in the United States (23, 24). However, integrating the WSCC model into school wellness policies and broader school efforts has been challenging. Here, we discuss five key challenges to WSCC implementation: (a) the complexity of the WSCC model; (b) a lack of resources to add anything new; (c) the problem of reactive policies; (d) consideration of implementation determinants, and (e) the need for leadership to be highly invested and engaged in implementation.

The first challenge is that the WSCC model is complex. It encompasses 10 domains of school wellness, and a key feature is that wellness efforts are coordinated across multiple members of the school and community system. The initial response of many schools is to add or delete language within existing policy structures to meet new requirements. However, this can cause schools to lose sight of the overarching goals of whole child development. To address this challenge, we recommend that schools use the strategy of Backwards Design (25), a curriculum design framework that begins by identifying desired outcomes (e.g., positive whole child development) and then determining the indicators of this outcome that would represent success. Next, initiatives are designed to (a) lead to the goal and (b) enable synergistic effects through the coordination and integration of policies and practices across WSCC domains (26). With this insight, we can organize our practices into an integrated continuum that ensures that all students have opportunities to maximize positive outcomes across developmental domains. Centering the desired goal in policy planning and decision-making offers one opportunity for strengthening the integration of the WSCC model into schools.

A second challenge facing schools is that they may lack the resources needed to add any new initiatives. Therefore, another opportunity for strengthening the integration of the WSCC model is to consider de-implementation (27). Schools need to balance their implementation efforts with available resources and select effective, efficient, and sustainable policies and practices. De-implementing policies or practices that are not generating intended outcomes can be a valuable way to free up resources for more promising work.

As noted earlier, a third challenge is that policies have historically been enacted in response to specific events or circumstances. When this happens, focus on the desired goal (e.g., whole child development) can be lost. For example, bullying policies often detail steps taken in response to reports of bullying, but do not explicate efforts to prevent bullying in school (28). In this case, a focus on promoting a safe environment along with procedures taken in response to bullying may be more effective in reducing bullying. Thus, taking a step back to identify broader goals related to whole child development can inform more transformative policy.

Evaluating the potential for efficient, effective, and sustainable implementation before taking on new initiatives is critical (27). Key questions addressing implementation determinants include: Is there data indicating the need for this initiative? Does it align with community values and school, district, or state priorities? Do we have the resources needed to successfully implement? Do staff have positive attitudes toward implementation and the knowledge and skills needed to implement? If any of these pieces are missing, it may be wise to strengthen these prerequisites for implementation. Otherwise, barriers to implementation are likely to arise and efforts to promote whole child development may be thwarted. Congruence is critical to successful and sustainable implementation.

Finally, strong leadership is key to successful and sustained implementation (29–31). Leaders set the tone, expectations, and climate for the implementation of any new policy. Several leadership theories point to the mechanisms through which leaders support implementation (29). The Full-Range Leadership Model (FRL), for example, highlights leaders’ potential to create a shared vision and positive work environment that emotionally and intellectually engages staff (32). Implementation leadership theory suggests that leaders achieve more positive outcomes when they are proactive, supportive, knowledgeable, and perseverant in their implementation efforts (33). Lastly, theories of implementation climate suggest that implementation is more successful when staff perceives the implementation of new practices as expected, supported, and rewarded in their setting (34, 35). The commitment, participation, and enthusiasm of leaders at multiple levels (e.g., school and district) further support a positive implementation climate. Thus, leaders desiring to implement or adjust WSCC-aligned policy should carefully examine their capacities to create a shared vision, actively participate in the work, and reinforce staff progress toward implementation; each of these contributes to the likelihood of successful and sustainable policy implementation.

## 5. Resources to advance use of the Whole School, Whole Community, Whole Child model

Several tools exist to support schools in strengthening their attention to the WSCC model in their policies. To score nutrition and physical activity policies, districts can use the Wellness School Assessment Tool (WellSAT), a quantitative self-assessment measure developed by the Rudd Center for Food Policy and Health (36). This measure has been updated three times and is used extensively by school districts to comply with the current requirement to complete a triennial assessment of their wellness policies (37).

To help school districts address their policies relevant to all 10 domains of the WSCC model, researchers from the Collaboratory on School and Child Health collaborated with the Rudd Center to create the WellSAT WSCC. The development process included item identification based on key concepts and best practice recommendations, expert review of the draft measure, cognitive pre-testing, development of scoring criteria, and pilot testing (38). This measure has been used to assess a sample of school district policies in Connecticut (39), and findings suggest that policy coverage of the WSCC model varies by domain and is often fragmented. The benefit of having a quantitative measure to assess district, state, or national policies is that it provides a structured process for districts to examine how their current policies align or are missing elements of the WSCC model and to document change over time.

To further support schools in the process of implementation, we have recently released the WSCC Policy Blueprint (27). The WSCC Policy Blueprint aims to support schools’ planful integration of the WSCC model into policy, including school wellness policies. The blueprint leads school and district leaders through 10 activities aimed at taking stock of current WSCC-related policies and practices (i.e., Exploring Context), identifying priority areas for strengthening (i.e., Evaluating Directions), and planning for successful adoption of policy changes (i.e., Establishing Actions).

The blueprints use the principles of Backwards Design discussed earlier (25). School personnel first identify their primary goals in strengthening policies to focus on whole child development. They are then provided resources to evaluate current policies for alignment with the WSCC model and introduced to opportunities for strengthening policies. The blueprint concludes with action planning for identified changes that have the highest urgency and readiness. The WSCC Policy Blueprint can be freely accessed at https://csch.uconn.edu/wscc-in-process/. Accompanying materials include the WSCC Practice Blueprint, a parallel guide for implementing coordinated and integrated WSCC practices, and several briefs on the WSCC model, individual domains, and the development of the WellSAT WSCC. These resources can all be accessed at no cost at https://csch.uconn.edu/.

## 6. Conclusion

When implemented effectively, traditional nutrition and physical activity school wellness policies can create substantially healthier school environments where children eat and play. In 2006, the top-down approach of a federal mandate to create these policies was needed to ensure that every school district focused on how they could be part of a national effort to reverse the trend of childhood obesity. Local attention was required to build momentum for state and then federal action (40). Local wellness policies were a key contributor to the significant transformation we have seen in the school food environment in the United States since 2006. However, this was yet another example of a policy that was created in reaction to a crisis instead of a proactive effort to promote a desired outcome.

Today, we can reconsider and broaden the concept of a school wellness policy. The WSCC model and the tools developed to support its implementation provide a structure for school districts to assess where they are in their current policies and practices. Using the principles presented above, schools can re-examine their policies to proactively promote whole child development.

## Data availability statement

The original contributions are included in the article. Further inquiries can be directed to the corresponding author.

## Author contributions

All authors listed have made a substantial, direct, and intellectual contribution to the work and approved it for publication.

## Conflict of interest

The authors declare that the research was conducted in the absence of any commercial or financial relationships that could be construed as a potential conflict of interest.

## Publisher’s note

All claims expressed in this article are solely those of the authors and do not necessarily represent those of their affiliated organizations, or those of the publisher, the editors and the reviewers. Any product that may be evaluated in this article, or claim that may be made by its manufacturer, is not guaranteed or endorsed by the publisher.
